# Neutrophil Elastase Deficiency Ameliorates Myocardial Injury Post Myocardial Infarction in Mice

**DOI:** 10.3390/ijms22020722

**Published:** 2021-01-13

**Authors:** Yukino Ogura, Kazuko Tajiri, Nobuyuki Murakoshi, DongZhu Xu, Saori Yonebayashi, Siqi Li, Yuta Okabe, Duo Feng, Yuzuno Shimoda, Zoughu Song, Haruka Mori, Zixun Yuan, Kazutaka Aonuma, Masaki Ieda

**Affiliations:** Department of Cardiology, Faculty of Medicine, University of Tsukuba, Tsukuba 305-8575, Japan; ogura.yukino.sj@alumni.tsukuba.ac.jp (Y.O.); n.murakoshi@md.tsukuba.ac.jp (N.M.); xu_dongzhu@md.tsukuba.ac.jp (D.X.); syonebayashi789@gmail.com (S.Y.); l.siqi@outlook.com (S.L.); yokabe0211@gmail.com (Y.O.); fengduoryu@outlook.com (D.F.); s2021402@s.tsukuba.ac.jp (Y.S.); s2030407@s.tsukuba.ac.jp (Z.S.); s1711822@s.tsukuba.ac.jp (H.M.); s1930477@s.tsukuba.ac.jp (Z.Y.); kaonuma@md.tsukuba.ac.jp (K.A.); mieda@md.tsukuba.ac.jp (M.I.)

**Keywords:** neutrophil elastase, myocardial infarction, apoptosis, remodeling, neutrophil, sivelestat

## Abstract

Neutrophils are recruited into the heart at an early stage following a myocardial infarction (MI). These secrete several proteases, one of them being neutrophil elastase (NE), which promotes inflammatory responses in several disease models. It has been shown that there is an increase in NE activity in patients with MI; however, the role of NE in MI remains unclear. Therefore, the present study aimed to investigate the role of NE in the pathogenesis of MI in mice. NE expression peaked on day 1 in the infarcted hearts. In addition, NE deficiency improved survival and cardiac function post-MI, limiting fibrosis in the noninfarcted myocardium. Sivelestat, an NE inhibitor, also improved survival and cardiac function post-MI. Flow cytometric analysis showed that the numbers of heart-infiltrating neutrophils and inflammatory macrophages (CD11b^+^F4/80^+^CD206^low^ cells) were significantly lower in NE-deficient mice than in wild-type (WT) mice. At the border zone between intact and necrotic areas, the number of terminal deoxynucleotidyl transferase dUTP nick end labeling (TUNEL)-positive apoptotic cells was lower in NE-deficient mice than in WT mice. Western blot analyses revealed that the expression levels of insulin receptor substrate 1 and phosphorylation of Akt were significantly upregulated in NE-knockout mouse hearts, indicating that NE deficiency might improve cardiac survival by upregulating insulin/Akt signaling post-MI. Thus, NE may enhance myocardial injury by inducing an excessive inflammatory response and suppressing Akt signaling in cardiomyocytes. Inhibition of NE might serve as a novel therapeutic target in the treatment of MI.

## 1. Introduction

Ischemic cardiovascular diseases, particularly acute myocardial infarction (MI), are one of the leading causes of mortality worldwide [1]. In the past few decades, with the advancement and innovation of pharmacological and interventional therapies, the acute mortality rate of MI has reduced remarkably [2]. Nevertheless, heart failure and life-threatening arrhythmias due to cardiac adverse remodeling remain challenges in clinical practice [3]. Following an MI, an inflammatory response is directed to the site of injury, resulting in structural and biochemical changes that can lead to cardiac remodeling [4].

Neutrophils are one of the major leukocytes involved in the inflammatory response to MI. During the first hours post-MI, neutrophils massively infiltrate into the infarct area [5]. Neutrophil elastase (NE) is a serin protease that is rapidly secreted extracellularly from azurophilic granules upon activation [6]. NE plays an important role in neutrophil-mediated bacterial killing. NE damages directly invading pathogens and fine-tunes the host inflammatory response for better pathogen eradication and its associated inflammation [7]. It has also been extensively documented that NE is involved in tissue destruction and inflammation, which characterize several noninfectious diseases, such as arthritis and respiratory diseases [8]. In contrast, the role of NE in heart diseases is less clear. Patients with acute MI have been reported to have higher plasma concentrations of NE [9]. However, the role of NE in the pathogenesis of MI remains unknown.

## 2. Results

### 2.1. NE Expression in MI Hearts

**Figure 1:**
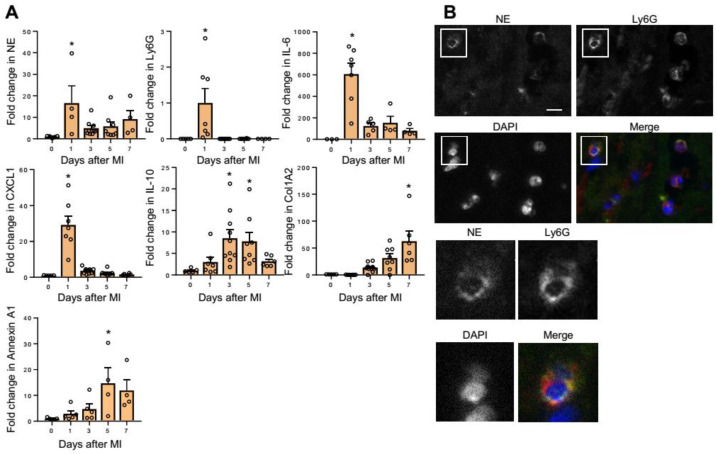
Neutrophil elastase (NE) expression peaked on day 1 post-myocardial infarction (MI) in infarcted hearts. (**A**) qRT-PCR analysis in hearts on day 0 (prior to the induction of MI) and on days 1, 3, 5, and 7 post-MI. Results are presented as mean ± SEM, *n* = 4–9 each, * *p* < 0.05 vs. day 0. (**B**) Representative immunofluorescence images of wild-type (WT) heart sections on day 1 post-MI. NE (yellow), Ly6G (red), and nuclei (blue). The lower panels show a higher magnification of the white boxes in the upper panels. Scale bar = 20 μm.

First, we investigated the gene expression of NE in the heart post-MI. The messenger RNA (mRNA) expression level of NE was significantly upregulated on day 1 post-MI (Figure 1A). This increase was consistent with an increase in the neutrophil marker lymphocyte antigen 6 complex, locus G (Ly6G) (Figure 1A). The mRNA levels of interleukin (IL)-6 and a neutrophil chemoattractant chemokine, C-X-C motif ligand (CXCL)1, also peaked on day 1, whereas genes related to resolution of inflammation (IL-10 and Annexin A1) and genes involved in tissue repair (type I collagen (Col1a2)) peaked at a later phase (Figure 1A). Immunofluorescence revealed that many NE-producing Ly6G-positive neutrophils infiltrated at the borders between the intact myocardial tissues and necrotic areas on day 1 post-MI (Figure 1B). These results indicate that infiltration of NE-expressing neutrophils into the myocardial tissue increased on day 1 post-MI, which is the acute phase of inflammation in MI.

### 2.2. NE Deficiency Improves Cardiac Function Post-MI

**Figure 2:**
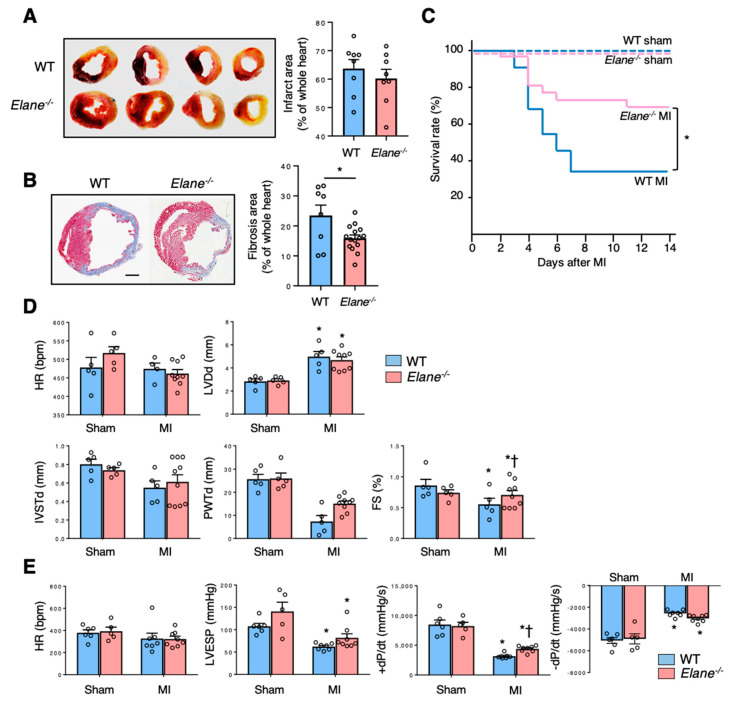
NE deletion improved survival and cardiac function post-MI. (**A**) Twenty-four hours post-MI, the hearts were removed and stained with 2,3,5-triphenyltetrazolium chloride for measurement of infarct area. Viable parts of the heart appear red, while the infarct area appears white. The bar graph represents quantification of infarct area as a percentage of the whole heart. Results are presented as mean ± SEM, *n* = 8 each. (**B**) Representative Masson’s trichrome staining images of the hearts from WT and *Elane*^−/−^ mice on day 7 post-MI. The bar graph shows quantification of fibrosis area as a percentage of the whole heart. Results are presented as mean ± SEM, *n* = 8–15 each, * *p* < 0.05. (**C**) Kaplan–Meier survival analysis 14 days post-MI or sham operation. Blue line indicates WT with MI (*n* = 22), red line indicates *Elane*^−/−^ with MI (*n* = 26), dotted blue line indicates WT with sham operation (*n* = 8), and dotted red line indicates *Elane*^−/−^ with sham operation (*n* = 7). * *p* < 0.05 by log rank. (**D**) Bar graphs represent echocardiographic parameters from the indicated mice. Results are presented as mean ± SEM, *n* = 5–9 each, * *p* < 0.05 vs. sham and # *p* < 0.05 vs. WT. (**E**) Bar graphs represent hemodynamic parameters 14 days post-MI. Results are presented as mean ± SEM, *n* = 5–8 each, * *p* < 0.05 vs. sham and † *p* < 0.05 vs. WT.

In order to investigate the role of NE in the pathophysiology of MI, we compared several cardiac parameters between WT and NE-deficient (*Elane*^−/−^) mice post-MI induction. First, we confirmed that the infarct size was similar between WT and *Elane*^−/−^ mice post-MI induction (Figure 2A). However, the extent of fibrosis was lesser in *Elane*^−/−^ hearts, as compared to WT hearts (Figure 2B). The survival rate of the *Elane*^−/−^ MI group was significantly higher than that of the WT MI group (Figure 2C). Echocardiographic examination and cardiac catheterization experiments revealed that *Elane*^−/−^ mice had improved cardiac function (preserved fractional shortening (FS) and increased +dP/dt), as compared to WT mice (Figure 2D,E). These results suggest that NE increases fibrosis size and exacerbates cardiac function post-MI.

### 2.3. NE Deficiency Suppresses Excessive Inflammation Post-MI

**Figure 3:**
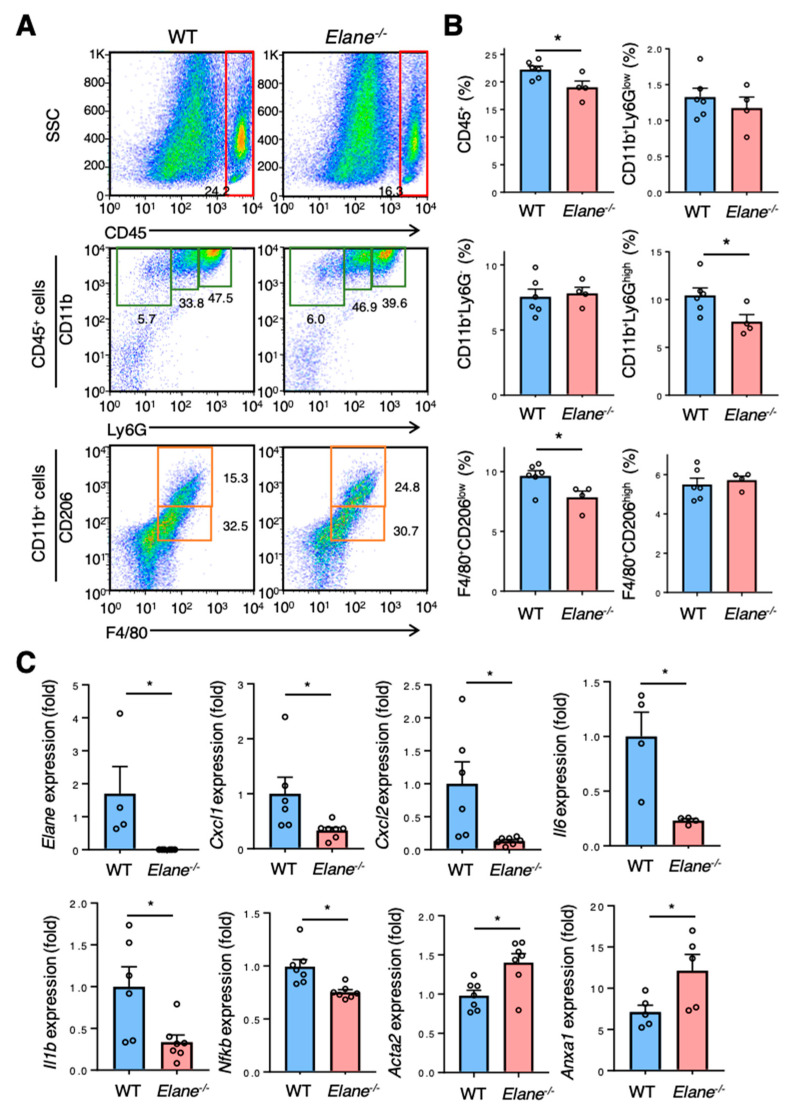
NE deletion suppressed excessive inflammation post-MI. (**A**) Representative flow cytometric plots showing CD45^+^ leucocytes, CD11b^+^Ly6G^low^ monocytes, CD11b^+^Ly6G^-^ macrophages, CD11b^+^Ly6G^high^ neutrophils, CD206^-^F4/80^+^ pro-inflammatory macrophages, and CD206^+^F4/80^+^ anti-inflammatory macrophages in WT and *Elane*^−/−^ hearts on day 3 post-MI. (**B**) Quantification of each inflammatory cells as a percentage of live cells from WT and *Elane*^−/−^ hearts on day 3 post-MI. Results are presented as mean ± SEM, *n* = 4–6 each, * *p* < 0.05. (**C**) mRNA expression of inflammatory, anti-inflammatory, and fibrotic markers in hearts from WT and *Elane*^−/−^ mice on day 3 post-MI. Results are presented as mean ± SEM, *n* = 6–8 each, * *p* < 0.05.

To examine the effect of NE on inflammatory cellular infiltration post-MI, flow cytometric analyses were performed on day 3 post-MI surgery. There was significantly less infiltration of inflammatory cells, such as CD45^+^ leukocytes, CD11b^+^Ly6G^+^ neutrophils, and F4/80^+^CD206^low^ inflammatory macrophages in the hearts of *Elane*^−/−^ MI mice than in the hearts of WT MI mice (Figure 3A,B). We then investigated the effect of NE on the mRNA levels of cytokines and chemokines in infarcted hearts. *Elane*^−/−^ mice had significantly reduced mRNA levels of the inflammatory cytokines (IL-1β, IL-6, CXCL1, CXCL2, and nuclear factor kappa B; Figure 3C) and increased gene expression levels of AnnexinA1 (related to resolution of inflammation) and genes involved in tissue repair (α-smooth muscle actin (ACTA2)) (Figure 3C). These results suggest that NE activates inflammatory responses and inhibits the resolution of inflammation and tissue repair.

### 2.4. NE Deficiency Reduces Cardiomyocyte Apoptosis by Activating Insulin/Akt Signaling

**Figure 4:**
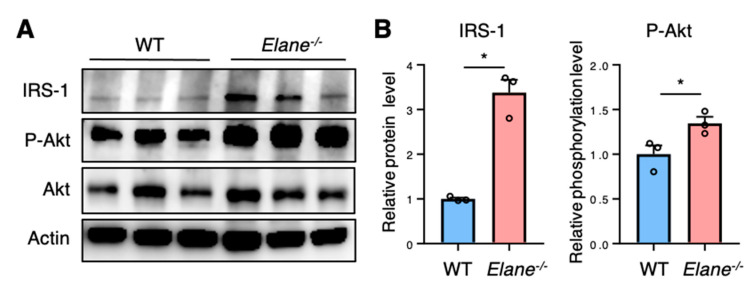
NE deletion enhanced Akt signaling post-MI. (**A**) Representative images of western blots for IRS-1, P-Akt, Akt, and Actin from WT and *Elane*^−/−^ hearts on day 3 post-MI. (**B**) The protein levels of IRS-1 were quantified using densitometry analysis and normalized to the levels of actin, while the levels of Akt phosphorylation were quantified and normalized to the levels of total Akt protein. Results are presented as mean ± SEM, *n* = 3 each, * *p* < 0.05. P-Akt, phosphorylated Akt.

The insulin-driven phosphoinositide 3-kinase (PI3K)/Akt pathway plays a central role in preventing apoptosis in cardiomyocytes [10]. Active insulin receptor recruits and phosphorylates insulin receptor substrate (IRS) proteins, which in turn activate the PI3K/Akt pathway [10]. Previous reports have shown that extracellular NE can gain access to the intracellular space and mediate degradation of IRS-1 [11,12]. Therefore, we performed western blotting to investigate whether NE affects Akt signaling via IRS-1 degradation in the heart post-MI. The protein levels of IRS-1 were lower in WT MI hearts than in *Elane*^−/−^ MI hearts (Figure 4A,B). In addition, the phosphorylation levels of Akt were lower in WT MI hearts than in *Elane*^−/−^ MI hearts (Figure 4A,B). These results suggest that NE deficiency might improve cardiomyocyte survival by increasing insulin/Akt signaling post-MI.

**Figure 5:**
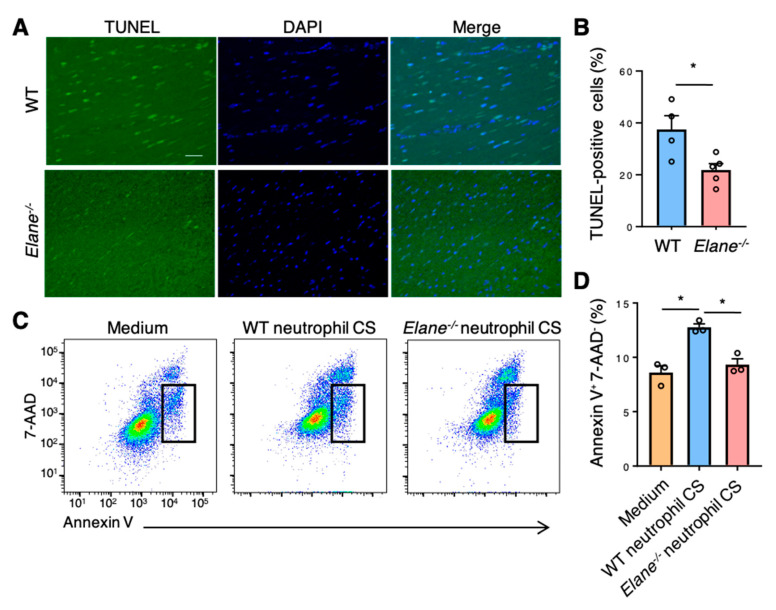
NE deficiency reduced cardiomyocyte apoptosis in vivo and in vitro. (**A**) Representative TUNEL staining images of WT and *Elane*^−/−^ heart sections on day 1 post-MI. Scale bar = 50 μm. (**B**) The bar graph shows the TUNEL-positive cells as a percentage of DAPI-positive cells in WT and *Elane*^−/−^ hearts on day 1 post-MI. Results are presented as mean ± SEM, *n* = 5, * *p* < 0.05. (**C**) Representative flow cytometric plots showing Annexin V^+^ and 7-AAD^−^ apoptotic HL-1 cells. HL-1 cells were cultured in the CS of WT or *Elane*^−/−^ neutrophils. Results are presented as mean ± SEM, *n* = 3 each, * *p* < 0.05. CS, culture supernatant.

We next performed terminal deoxynucleotidyl transferase dUTP nick end labeling (TUNEL) assay to investigate the effect of NE on cardiomyocyte apoptosis post-MI. There were more TUNEL-positive apoptotic cells in the border zones between the intact myocardial tissues and necrotic areas in WT mice than in *Elane*^−/−^ MI mice (Figure 5A,B). Moreover, we assessed the effect of NE on apoptosis in vitro using a cardiomyocyte cell line (HL-1). Flow cytometric analyses revealed that the culture supernatant of WT neutrophils induced more apoptosis (Annexin V^+^7-AAD^−^ cells) in HL-1 cells than in the *Elane*^−/−^ neutrophil culture supernatant (Figure 5C,D). These findings suggest that NE may cause apoptosis in cardiomyocytes through IRS-1 degradation and downregulate the downstream Akt signaling post-MI.

### 2.5. NE Inhibitor Improves Survival and Cardiac Function Post-MI

**Figure 6:**
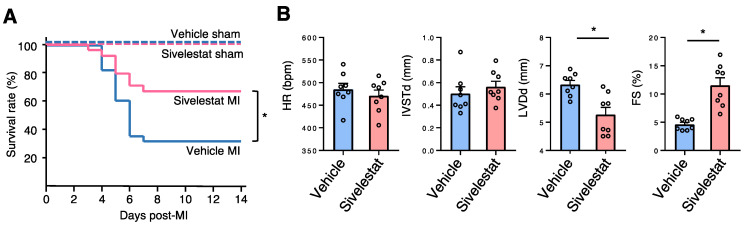
Sivelestat improved survival and cardiac function post-MI. (**A**) Kaplan–Meier survival analysis 14 days post-MI treatment with sivelestat or vehicle. Blue line indicates vehicle treatment with MI (*n* = 28), red line indicates sivelestat treatment with MI (*n* = 24), dotted blue line indicates vehicle treatment with sham operation (*n* = 5), and dotted red line indicates sivelestat treatment with sham operation (*n* = 7). * *p* < 0.05 by log rank. (**B**) Bar graphs represent echocardiographic parameters from the indicated mice. Results are presented as mean ± SEM, *n* = 8 each, * *p* < 0.05.

Finally, we investigated the therapeutic potential of an NE inhibitor, sivelestat. We set the sivelestat dose (100 mg/kg/day, daily) according to that mentioned in a previously published study in which this drug was used to prevent asbestos-induced lung fibrosis in mice [13]. The survival rate of the sivelestat MI group was significantly higher than that of the vehicle MI group (Figure 6A). Echocardiography revealed that the sivelestat MI group displayed smaller left ventricular size and better cardiac contraction (Figure 6B). These results suggest that sivelestat may have therapeutic potential for MI.

## 3. Discussion

**Figure 7:**
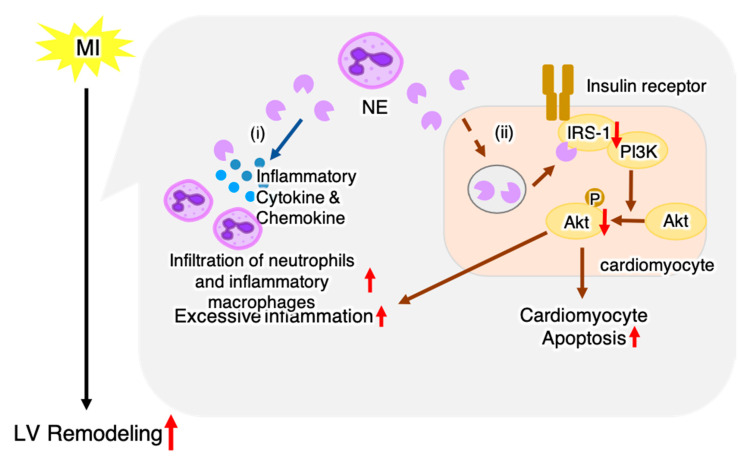
Schematic illustration showing a hypothetical mechanism of NE-mediated LV remodeling post-MI. Post-MI, NE-producing neutrophils infiltrate the heart; our data suggest that NE exacerbates LV remodeling through two pathways: (**i**) NE released from neutrophils induces excessive inflammation, and (**ii**) NE decreases Akt signaling via degradation of IRS-1 leading to cardiomyocyte apoptosis and excessive inflammation. The red arrow indicates the effect of NE.

In this study, we revealed a novel role of NE in MI. NE deficiency improved survival and cardiac function, in addition to decreasing fibrosis and apoptosis post-MI. In NE-deficient mice, there was a reduction in the number of heart-infiltrating inflammatory cells and expression of inflammatory-related genes, while the expression levels of genes related to resolution of inflammation and tissue repair increased. NE deficiency protected from a decrease in IRS-1 levels and increased Akt signaling in the heart post-MI. Our data suggest that NE enhanced myocardial injury by inducing an excessive inflammatory response and suppressing Akt signaling in cardiomyocytes, thereby worsening the prognosis post-MI (Figure 7).

Previous studies have shown that post-MI inflammation is essential for the healing process, but excessive inflammation is associated with progression of left ventricular (LV) remodeling and worse prognosis [14,15]. We found that, compared to WT mice, NE-deficient mice displayed diminished heart inflammation, as evidenced by decreased number of heart-infiltrating inflammatory cells and a significantly lower mRNA expression level of proinflammatory genes within the hearts upon NE deficiency. These results suggest that NE is involved in excessive inflammation post-MI. Accumulated evidence has identified NE as a mediator that enhances inflammation through multiple mechanisms. NE upregulates neutrophil chemokines such as IL-8 [16], proteolytically activates chemokines such as IL-1α or IL-33 [17], enhances proteolysis of cell surface-bound cytokine receptors or cytokine-binding proteins, and activates specific cell surface receptors (such as toll-like receptor 4) [18,19]. We found that cardiac expression levels of multiple proinflammatory cytokines and chemokines (e.g., IL-1β, IL-6, CXCL1, and CXCL2) were significantly decreased in NE-deficient mice post-MI (Figure 3C). These findings indicate that NE promotes inflammatory response by activating these proinflammatory cytokines, which is consistent with previously reported findings. In addition, NE deficiency has been reported to suppress neutrophil adhesion and migration [20]. We found that CD11b^+^Ly6G^high^ neutrophil infiltration was significantly decreased in NE-deficient mice (Figure 3A), suggesting that NE may directly affect neutrophil infiltration in the heart post-MI. In this study, we found that NE deletion increased P-Akt levels in the heart post-MI (Figure 4). Akt has been reported to reduce inflammatory responses in various cells, including cardiomyocytes [21,22,23]. Thus, a decrease in Akt signaling induced by NE may also contribute to excessive inflammation post-MI.

Cardiomyocyte apoptosis is a key feature of pathological cardiac remodeling post-MI, and depletion of cardiomyocytes at the border zone between infarcted and noninfarcted areas causes structural and functional damage, resulting in heart failure [24,25]. Our current data indicated that NE deficiency protects against apoptosis in post-MI hearts. In cardiomyocytes, insulin-driven Akt signaling is important for protection from apoptosis [26]. Insulin binds to insulin receptor expressed in myocardial cells, which leads to Akt activation via phosphorylation of IRS-1 [27]. Previous reports have shown that extracellular NE can gain access to the intracellular space and mediate degradation of IRS-1 [11,12]. Our in vivo data showed that NE deficiency led to increased IRS-1 protein levels and P-Akt in post-MI hearts (Figure 4). These data suggested that degradation of IRS-1 was suppressed upon NE deficiency, which in turn activated Akt signaling and protected cardiomyocytes from apoptosis. In this study, we did not test the effect of sivelestat in vitro. Sivelestat is an acylation inhibitor that inhibits enzyme activity by forming an acylation complex with the active center of neutrophil elastase [28]. Therefore, sivelestat is expected to inhibit NE activity and suppress cardiomyocyte apoptosis in vitro as well.

In addition to NE deletion, administration of an NE pharmacological inhibitor, sivelestat, also improved survival and cardiac function post-MI (Figure 6). Our findings suggest that NE inhibitors may be useful in suppressing excessive inflammation and apoptosis and preventing LV remodeling post-MI.

## 4. Materials and Methods

### 4.1. Mice

C57BL/6J mice were purchased from Clea Japan (Tokyo, Japan). NE-deficient (*Elane*^−/−^) mice were originally backcrossed onto the C57BL/6J genetic background for 12 generations and then bred in our laboratory. All mice used for the experiments were male. All animal experiments were approved by the Institutional Animal Experiment Committee of the University of Tsukuba (approved number: 13402, approved date: 1 June 2013) and conformed to the NIH Guide for the Care and Use of Laboratory Animals.

### 4.2. MI Model

MI was induced in approximately 10–12 weeks old male mice that weighed at least 25 g. The classical MI-induction method was followed, as previously described [29,30]. Briefly, mice were anesthetized by intraperitoneal injection of ketamine/xylazine (100–120 mg/kg body weight for ketamine and 7–8 mg/kg body weight for xylazine), intubated, and connected to a ventilator (Mouse Ventilator Minivent Type 845; Harvard Apparatus, Holliston, MA, USA). The chest cavity was opened via left thoracotomy to expose the heart, allowing the left anterior descending coronary artery to be visualized by eye or magnifying glasses, which was then permanently ligated with a 7–0 nylon suture, at the site of its emergence from the left atrium. Complete occlusion of the vessel was confirmed by the presence of myocardial blanching in the perfusion bed. Mice that died during recovery from anesthesia were excluded from the analysis. For sham mice, the same MI induction procedure was performed, but without carrying out the left anterior descending artery ligation. Sivelestat was obtained from ONO Pharmaceutical Co. Ltd., Osaka, Japan. To test the effect of sivelestat in MI, the mice were intraperitoneally injected with sivelestat (100 mg/kg/day) or vehicle (saline) once daily for 7 days, starting from the day of the MI surgery.

### 4.3. Survival Analysis

A total of 127 mice were used in the survival analysis (WT sham, *n* = 8; KO sham, *n* = 7; WT MI, *n* = 22; *Elane*^−/−^ MI, *n* = 26; vehicle sham, *n* = 5; sivelestat sham, *n* = 7; vehicle MI, *n* = 28; sivelestat MI, *n* = 24). To reduce the suffering of the mice, we set humane endpoints to decide when to euthanize the mice, as previously described [29]. The humane endpoints included body temperature and physical activity that were significantly worse than the active mice, including a decrease or no increase in a few hours; or the mice did not respond to three intermittent stimulations in half an hour, or the respiratory rate of mice was abnormally rapid or slow, or there was rapid/progressive weight loss (a 20% loss of body weight). Mice were euthanized by lethal intraperitoneal injection of sodium pentobarbital (200 mg/kg) or CO_2_ inhalation when they reached these endpoints to avoid further distress. Only the animals reaching the endpoint before the evaluation at the end of the study were considered valid and used in the survival analysis. The mice were cared for by trained staff and were monitored every 12 h post-surgery. After 14 days, all mice that survived were euthanized by lethal intraperitoneal injection of sodium pentobarbital (200 mg/kg) or CO_2_ inhalation.

### 4.4. Echocardiography

Transthoracic echocardiography was performed with a Vevo^®^ 2100 instrument (Fujifilm Visual Sonics, Tokyo, Japan) equipped with an MS-400 imaging transducer. Isoflurane induction was performed in an induction box with 3% isoflurane in pure medical oxygen. After the mouse’s righting reflex waned, it was fixed in the supine position on a heating pad to maintain normothermia, followed by placement of electrocardiographic limb electrodes. Anesthesia was maintained with 1% isoflurane. Echocardiography parameters, including heart rate, interventricular septum thickness at end-diastole, left ventricular posterior wall in diastole, left ventricular diameter at end-diastole, and FS were measured.

### 4.5. Histopathological and Immunohistochemical Examination

The hearts were fixed with 4% paraformaldehyde, embedded in paraffin wax, sectioned into 3-μm thick slices, and stained with Masson’s trichrome. Fibrosis area was measured and analyzed using ImageJ analysis software (version 1.53; National Institutes of Health, Bethesda, MD, USA). For immunohistochemical examination, deparaffinization and antigen activation of the sections were performed, followed by incubation of the sections with rabbit polyclonal anti-NE antibodies (ab668672; Abcam, Cambridge, UK) or mouse anti-Ly6G antibodies (127,601; BioLegend, San Diego, CA, USA) at 4 °C overnight. Next, the sections were sufficiently rinsed in phosphate-buffered saline (PBS) and incubated with Alexa Fluor^®^ 633-labeled goat antirabbit IgG (Thermo Fisher Scientific, Waltham, MA, USA) or Alexa Fluor^®^ 546-labeled goat antimouse IgG (Thermo Fisher Scientific) at room temperature for 2 h. After rinsing in PBS and mounting onto slides using mounting medium with DAPI, fluorescent images of the slides were captured using a digital fluorescence microscope (Biozero BZ-X700; Keyence, Osaka, Japan).

### 4.6. Infarct Size Evaluation

To evaluate the infarct size on day 1 post-MI, the hearts were weighed and frozen at −80 °C. The frozen hearts were cut transversely into 1-mm thick slices using a Mouse Heart Slicer Matrix (Zivic Instruments, Pittsburgh, PA, USA) and stained with 2% 2,3,5-triphenyltetrazolium chloride in PBS (pH 7.4) for 20 min in a 37 °C water bath. After fixation for 4 to 6 h in 10% neutral buffered formaldehyde, both sides of each slice were photographed. The viable myocardium was stained brick red, while the infarct tissues appeared pale white. Infarct and LV area were measured using automated planimetry with ImageJ software. The infarct size was then expressed as a percentage of the total LV area.

### 4.7. TUNEL Assay

Apoptotic cells were assessed using TUNEL staining with a Click-iT^®^ Plus TUNEL assay kit (Thermo Fisher Scientific), according to the manufacturer’s instructions. After deparaffinization and antigen activation, the sections were incubated with the TUNEL reaction mixture (terminal deoxynucleotidyl transferase and nucleotides) for 1 h at 37 °C in the dark. The slides were then rinsed three times with PBS and observed under a fluorescence microscope. The percentage of apoptotic cells was determined by counting the total number of cells double-positive for fluorescein and DAPI using ImageJ analysis software.

### 4.8. Flow Cytometry

For the evaluation of infiltration of immune cells into hearts post-MI, the excised hearts were minced with fine scissors into 1–2 mm^3^ pieces. The minced heart tissue was transferred into a tube containing enzyme solution (200 units/mL collagenase II (Worthington, Lakewood, NJ, USA), 500 units/mL hyaluronidase type IV-S (Sigma-Aldrich, St. Louis, MO, USA), and 100 units/mL DNase I (Takara Biotec, Kusatsu, Japan) and dissociated using a gentleMACS™ Dissociator (Miltenyi Biotec, Bergisch Gladbach, Germany). The dissociated cells were stained directly using fluorochrome-conjugated mouse-specific antibodies and analyzed with a FACSVerse™ instrument (BD Biosciences, San Jose, CA, USA) using FlowJo version 10.2 software (BD Biosciences). Cells that did not stain with 7-AAD (TONBO Biosciences, San Diego, CA, USA) were deemed viable. The antibodies used were as follows: anti-CD45 (clone 30-F11; eBioscience, San Diego, CA, USA), anti-Ly6G (clone RB6-8C5; TONBO Biosciences), anti-CD11b (clone M1/70; eBioscience), anti-F4/80 (clone BM8.1; TONBO Biosciences), and anti-CD206 (clone C068C2; BioLegend). To evaluate apoptosis in vitro, HL-1 cells were stained with 7-AAD and FITC Annexin V (BD Biosciences).

### 4.9. Cell Culture of HL-1 Cells

Neutrophils were isolated from WT and *Elane*^−/−^ mice using a magnetic-activated cell sorting kit (Neutrophil Isolation Kit, Miltenyi Biotec) and cultured with DMEM containing 1% penicillin-streptomycin for 24 h. HL-1 cells were cultured with the culture supernatant of the neutrophils or DMEM medium for 24 h in a humidified incubator at 37 °C and 5% CO_2_.

### 4.10. Flow Cytometric Analysis of Cardiomyocyte Apoptosis

HL-1 cells were resuspended in Annexin V Binding Buffer (BioLegend). 100 µL of cell suspension were stained with 1 µL of FITC Annexin V (BioLegend) and 1 µL of 7-AAD Viability Staining Solution (BioLegend) and incubated for 15 min at room temperature in the dark. After adding 400 µL of Annexin V Binding Buffer (BioLegend), we analyzed cell apoptosis using flow cytometry.

### 4.11. RNA Extraction and Quantitative Reverse-Transcription Polymerase Chain Reaction

All hearts removed for qRT-PCR were snap-frozen and stored at −80 °C. For preparation of total RNA, the tissue was homogenized using a bead kit (MagNA Lyser Green Beads; Roche Diagnostics, Indianapolis, IN, USA), according to the manufacturer’s instructions. Total RNA from heart tissue and cultured cells was extracted using an RNeasy^®^ Fibrous Tissue Mini Kit (QIAGEN, Hilden, Germany), according to the manufacturer’s instructions. Complementary DNA was synthesized from 1 μg total RNA using a High-Capacity cDNA Reverse Transcription Kit (Applied Biosystems, Waltham, MA, USA). qRT-PCR analysis was performed using the LightCycler^®^ 480 system (Roche Applied Science, Penzberg, Germany) with a Universal Probe Library (Roche Applied Science). Hypoxanthine-guanine phosphoribosyltransferase RNA was used as an internal control. Gene expression values were calculated using the 2^−ΔCt^ method.

### 4.12. Western Blotting

Western blotting was performed as described previously [31]. In brief, isolated hearts were homogenized in PRO-PREP™ protein extraction solution (iNtRON Biotechnology, Inc. Kyungki-Do, Korea) and the supernatants obtained were used for Western immunoblotting. Appropriate volumes of the samples (10 μg) were mixed with an equal volume of sample buffer, heated at 95 °C for 5 min, and then subjected to sodium dodecyl sulfate-polyacrylaminde gel electrophoresis using 4–15% gradient polyacrylamide gels (Bio-Rad, Hercules, CA, USA). The proteins were transferred from gels to polyvinylidene difluoride membranes using semidry electroblotting. The blots were then blocked and incubated with the following primary antibodies: IRS-1 (1:500, #2382), P-Akt (Ser^473^) (1:1000, #9271), Akt (1:1000, #9272) (all purchased from Cell Signaling Technology Inc., Danvers, MA, USA), and mouse monoclonal β-actin (1:1000, C4) (sc-47778, Santa Cruz Biotechnology, Dallas, TX, USA). β-actin was used as a loading control because it is widely and consistently expressed in all types of eukaryotic cells, and the protein level is known to remain stable during experimental treatments. The blots were incubated with an appropriate secondary antibody, horseradish peroxidase (HRP)-conjugated goat antirabbit IgG (1:5000, ab6721, Abcam) or HRP-conjugated rabbit antimouse IgG (1:5000, ab97046, Abcam). Immunoreactions were visualized with an enhanced chemiluminescence method (ECL™ Prime Western Blotting Detection; GE Healthcare, Southeast, UK). Densitometric analysis was performed on scanned immunoblot images using the ImageJ analysis software. The ratios of densities of bands detected using phosphorylated antibodies to those detected using nonphosphorylated (total) antibodies or β-actin antibody were obtained from two independent measurements (*n* = 3 per group for each measurement).

### 4.13. Statistical Analysis

All data are expressed as mean ± standard error of the mean. Normality was verified using the Shapiro–Wilk test. Statistical analyses were performed using an unpaired two-tailed *t* test or Mann–Whitney *U* test for comparison of two groups. For multiple comparisons, one-way analysis of variance with a Newman–Keuls post hoc test or a Kruskal–Wallis analysis with a Steel–Dwass or Steel post hoc test was used. Survival distributions were estimated using the Kaplan–Meier method and compared using the log-rank test. *p* < 0.05 was considered statistically significant. All statistical analyses were performed using JMP software (SAS Institute).

## Data Availability

The data that support the findings of this study are available from the corresponding author (K.T.), upon reasonable request.

## Abbreviations

CD	cluster of differentiation
Col1a2	collagen type I alpha 2 chain
CS	culture supernatant
CXCL	C-X-C motif chemokine ligand
DAPI	4′,6-diamidino-2-phenylindole
FS	fractional shortening
HR	heart rate
IL	interleukin
IRS-1	insulin receptor substrate-1
IVSTd	interventricular septal in end-diastole
LV	left ventricular
LVDd	left ventricular diameter at end-diastole
LVESP	left ventricular end-systolic pressure
Ly6G	lymphocyte antigen 6 complex, locus G
MI	myocardial infarction
mRNA	messenger RNA
NE	neutrophil elastase
NFκB	nuclear factor kappa B
PBS	phosphate-buffered saline
PI3K	phosphoinositide 3-kinase
PWTd	left ventricular posterior wall in end-diastole
qRT-PCR	quantitative reverse transcription polymerase chain reaction
TTC	2,3,5-triphenyltetrazolium chloride
TUNEL	terminal deoxynucleotidyl transferase dUTP nick end labeling
WT	wild-type
